# Projected cancer burden, challenges, and barriers to cancer prevention and control activities in the state of Telangana

**DOI:** 10.1371/journal.pone.0278357

**Published:** 2023-07-14

**Authors:** Hemant Mahajan, Neha Reddy, N. G. Marina Devi, Usha Rani Poli, M Jayaram, Shailaja Tetali, G. V. S. Murthy, on behalf of the Telangana Cancer Control Study Group

**Affiliations:** 1 Department of Public Health, Indian Institute of Public Health, Hyderabad, Telangana, India; 2 Department of Clinical Research, London School of Hygiene & Tropical Medicine, London, United Kingdom; SRM Institute of Science and Technology, INDIA

## Abstract

**Background and aim:**

The Telangana cancer care program is a proactive, comprehensive initiative encompassing infrastructure development, human resource skilling and ensuring financial protection to those below poverty line. The broad aim of this exercise was to identify modalities to augment the Telangana State Cancer Control Plan to implement a sustainable comprehensive cancer care model for Telangana.

**Methods:**

We conducted in-depth interviews of stakeholders (17 patients and 25 health care providers) to identify barriers and challenges to access existing cancer care system in Telangana; calculated the magnitude of cancer and commensurate workload (in terms of visits to tertiary cancer care system for cancer management and human and equipment requirement) for the next 15 years (from 2022 to 2037). Using the anecdotal evidence and information from stakeholders’ interviews, we developed patient-journey funnels for oral, breast, and cervical cancer patients to highlight patient leakages at various levels of cancer care.

**Results:**

We estimated a 13%, 28%, and 44.7% increase in the number of new cancer cases and the resultant workload (number of visits to health care centre, chemotherapy sessions, radiotherapy sessions, surgeries, specialized human resources and equipment), for the year 2027, 2032, and 2037, respectively, compared to the year 2022. The stakeholders mentioned ‘delayed access’ to healthcare system as the main reason for the poor prognosis of patients. The common reasons cited for ‘delayed access’ were: poor cancer-literacy including prevailing myths and misconception, financial barriers, and rural residence. The patient journey funnel for cancer care revealed a major leakage from ‘screened-positive’ to ‘diagnosis confirmation’ step. The estimated patient leakage varied from ~70% to 90% from ‘screened-positive’ till ‘treatment completion’.

**Conclusion:**

In this study, we anticipated a steady increase in the number of new cancers cases and resultant workload for the state of Telangana from the year 2022 to 2037. This may further be accompanied with limited access or utilization of cancer care system. To manage this public health issue, government should take appropriate measures to improve cancer literacy at the community level as well as increase human resources and necessary equipment.

## Introduction

The burden of cancer is rising across all states in India at a variable pace [1,2]. This heterogeneity of cancer burden and/or health loss (across states) is due to differences in the population genetics, social development, lifestyle, and environment. Owing to the epidemiological transition [3] (defined as the ratio of all-age disability-adjusted life years (DALYs) due to communicable diseases versus those due to non-communicable diseases (NCDs) and injuries together), the greatest increase in crude cancer incidence rates has been observed in states with high epidemiological transitions (an increase in the proportion of disease burden attributable to NCDs) while the mortality-to-incidence ratio (defined as number of deaths divided by number of newly diagnosed cancer cases in a given year) is greater in the other regions [1]. It has been reported that the age-adjusted cancer DALYs vary by 2.6 times across the states in India, which emphasizes the need for contextualized state-specific interventions to handle the spiraling burden [1].

Telangana, one of the southern states in India, has experienced a recent surge in number of cancer cases with crude-cancer incidence rate of 72.6 (69.4, 77.3) and age-adjusted incidence rate of 88.7 (85.1, 94.0) per 100,000 in 2016 [1]. The state-specific crude cancer mortality-to-incidence ratio was estimated to be 0.70 for females and 0.81 for males, which indicates poorer survival especially among males [1]. Of all the five neighboring states, cancer-related mortality-to-incidence ratio among females as well as males is only lower than Andhra Pradesh and Odisha. Among all population-based cancer registries in India, Hyderabad district (the capital of Telangana) recorded the highest incidence rate for breast cancer (leading cause of cancer mortality for women in Telangana) - 48/100,000 [2]. Moreover, two thirds of the cases of triple negative breast cancer (aggressive form of breast cancer with poor prognosis) were reported among participants aged less than 50 years [4]. Among men, the cancers with highest prevalence in the Hyderabad district were mouth, lung, and the tongue cancers [2].

The problem of the high burden of cancer in Telangana is further complicated by inadequate healthcare infrastructure (such as inadequate human resources, equipment etc.) due to lack of state-run health institutes dedicated for cancer management especially at primary and secondary level [5,6]. This highlights a need to strengthen the healthcare system in Telangana to facilitate the comprehensive cancer care, especially in rural areas (which is unequally experiencing the cancer risk factors and preventable morbidity, mortality, and sufferings from cancers compared to their urban counterparts) [7,8]. Therefore, we conducted an assessment of the current control initiatives to identify potential public health value additions to the existing cancer care initiative of the state and strengthen it further. The broad aim of the initiative was to complement the excellent cancer care initiatives of the government of Telangana by building patient-centric pathways for comprehensive cancer care alongside developing academic and research capabilities in the state. The specific objectives were (a) To identify measures to- strengthen the Telangana state comprehensive cancer care plan contextualized to local priorities and learnings from global best practices/models; (b) To co-create tangible interventions for implementation at the state level; and (c) To develop a robust advocacy plan and engage with policy makers.

In this paper we are presenting (a) cancer-specific burden and the commensurate workload for the state of Telangana for next 15 years (from 2022 to 2037); and (b) The findings from the stakeholder interviews at different levels of health care system to explore their perspectives on barriers and facilitators for comprehensive cancer care services in the state.

## Materials and methods

### Study population

The work on the project was done between February and September 2021. The project was approved by the Institutional Ethics Committee of the Indian Institute of Public Health–Hyderabad (Approval no.: IIPHH/TRCIEC/228 /2020; Dated: 11-12-2020). Data was extracted from published and grey literature to assess the magnitude of cancers, the determinants and barriers to access services, skills and workload, technology innovations in the continuum of cancer care, patient journey, palliative care services and successful cancer care interventions in other states in India and other parts of the world.

### Assessment of magnitude of cancer

The estimated population of Telangana for 2022 to 2037 was retrieved from the ‘Report of the technical group on population projections, 2019’ [9]. For estimating the cancer-specific burden in Telangana, five-year prevalence of cancer in India (197.1 per 100000) as reported by Global Cancer Observatory, 2020 was considered (due to the absence of cancer-prevalence data for Telangana) [10]. New cancer cases per year from 2022 to 2037 was estimated using the estimates of cancer incidence (stratified by sex) from ICMR-NCDIR report on “Profile of Cancer & Related factor for the state of Telangana” [8]. Briefly, this report has used the data from the population based cancer registry, Hyderabad collected from the year 2014 to 2016 and calculated crude incidence rate and relative proportion of common cancers, separately for males and females. The authors have also estimated the number of new cancer cases for the year 2020 and 2025 using the crude incidence, age-specific cancer incidence for Telangana, and census data from the year 2001 and 2011 [8]. We used the projected cancer cases for the year 2020 and 2025 and calculated the relative annual percent change (APC) in new cancer cases separately for males and females. Using these relative APCs in new cancer cases (males: 2.4%; females: 2.58%), we calculated the expected number of new cancer cases from the year 2022 to 2037 for the state of Telangana.

We assumed the yearly cancer survival rate of 90% (conservative estimate) and cases surviving at the end of each year were estimated. These cases were considered to be the baseline for the subsequent year. For example, in the year 2022: at the beginning there were 74,356 cancer cases; the new estimated cancer cases are 50024; and yearly survival rate of 90%; Total cases surviving at the end of the year 2022 will be 111942. These 111942 cases surviving till the end of 2022 were considered as the prevalent cancer cases at the beginning of the year 2023.

To estimate the number of new cases for the common cancers including breast, cervical, oral, and lung cancer, we used the relative proportion (%) of these cancers to total cancer cases for the year 2022 [8]. Among females, relative proportion for the breast, cervical, oral, and lung cancers were 35.50%, 8.70%, 3.50%, and 4.10%, respectively. Among males, relative proportion for the oral and lung cancers were 21.21% and 10.90%, respectively. To project the numbers from these cancers for the next 15 years (2022 to 2037), we used the relative APC reported in the LANCET report. Among females, the relative APC for the breast, cervical, oral, and lung cancers reported are +1.58%, -1.65%, -0.0923%, and -0.42%, respectively [1]. Among males, the relative APC for the oral and lung cancers reported are -0.02% and -0.3%, respectively [1]. Survival rates for India were applied to determine magnitude of burden from each of these cancers in Telangana [11].

### Assessment of magnitude of cancer workload, demand for human resources and equipment

To understand the workload for managing different cancers, the magnitude of cancers alone will underestimate the workload, as cancer patients have to make a number of visits for the surviving period with cancer for treatment and follow up. Workload at the tertiary cancer centers was estimated based on the site and stage of a cancer. Data on relative proportion of cases diagnosed in early and advanced stages was extracted from the Report on the National Cancer Registry Programme—2020 [2] as well as the ‘Profile for Cancer & Related factors for the state of Telangana Report’ [8], which present information on stages at diagnoses for specific cancers from hospital-based cancer registries across India and Telangana, respectively. Average number of visits required during each phase (pre-and post-diagnosis) were determined based on in-depth interviews with key stakeholders (oncologists) from public as well as private hospitals.

• Work-load was estimated using the formula:
 ○ Work-load = New case **×** average visits per case during cancer management
• Demand for human resources (cancer specialists) and equipment for the respective years was estimated using the formula [12]:
 ○ No. = Estimated new cases in the respective year **×** Norm per 1000 new patients

### Assessment of chemotherapy, radiotherapy, and surgery requirements

Based on the estimated new cases in the respective years, cancer cases requiring chemotherapy, radiotherapy and surgery were calculated for three assumed scenarios: Scenario 1–55% of new cases requiring chemotherapy and/or radiotherapy and 45% of new cases requiring surgery; Scenario 2–60% of new cases requiring chemotherapy and/or radiotherapy and 40% of new cases requiring surgery; Scenario 3–65% of new cases requiring chemotherapy and/or radiotherapy; 35% of new cases requiring surgery. These scenarios were assumed based on the experiences shared by the Oncologists related to clinical extent of disease at presentation as well as the clinical extent of disease at presentation for cancers calculated from the Hospital-based registries across India [2]. More than 50% of the new cases of common cancers (oral, breast, cervical, lung, etc.) present in the loco-regional or distant metastasis stage (to the tertiary cancer care centers) [8].

- For each of the scenarios, total cases requiring chemotherapy was calculated using the formula:
  ○ Total cases requiring chemotherapy = [Estimated new cases in a given year **×** Percentage requiring chemotherapy]
- To calculate chemotherapy cycles per day, assuming six cycles are required per patient, the formula used was as follows:
  ○ Chemotherapy cycles per day = [New cases requiring chemotherapy in a given year **×** 6]/365
- Similarly, for estimating linear accelerators (LINACs) for radiotherapy sessions, we assumed 22 radiotherapy sessions per patient and 15,000 radiotherapy sessions per machine. The following formula was applied:
  ○ New LINACs for radiotherapy sessions in a given year = [Estimated new cases requiring radiotherapy in a given year **×** 22]/15000
- Onco-surgeries that are likely to be performed a month were estimated by applying the formula:
  ○ Onco-surgeries that are likely to be performed a month = [Estimated new cancer cases requiring surgery annually]/12

For the quantitative data such as cancer magnitude, workload, etc., we calculated proportion (as appropriate) and 95% confidence interval using Binomial Exact method using STATA version 14.2 (Stata Corp, College Station, TX, USA).

### In-depth interviews with stakeholders

In-depth interviews using unstructured questionnaires were conducted with various healthcare providers (n = 25) serving at different levels of healthcare system in different capacities. The in-depth interviews (Descriptive Phenomenological Method of Qualitative Analysis) were mainly conducted to explore stakeholders’ perspectives on barriers and facilitators of comprehensive cancer care services in the state. The sampling for in-depth interviews was purposive and until themes were saturated (no new relevant knowledge was being obtained from new participants.) For healthcare providers group, we identified oncologists, medical officers at primary health centers (PHC), community health centers (CHC) and district hospital (DH), staff-nurses, auxiliary nurse and midwife (ANMs), mid-level health provider (MLHP), palliative care physicians, district programme officer of National Program for the Prevention and Control of Cancer, Diabetes, Cardiovascular diseases and Stroke (NPCDCS), and administrative secretary of a non-governmental organization. We selected these participants with the prior assumption that they share the common characteristics of serving patients diagnosed with cancer and have the potential to provide relevant and diverse information for the study objectives.

We also conducted the in-depth interviews with 17 cancer patients to explore their perceptions about cancer and existing cancer case services in the state of Telangana. We purposively selected 17 patients with different sites of neoplasms and different stages from three tertiary cancer care centers in Hyderabad to retrieve diverse and comprehensive information.

In consultation with cancer experts, we developed the interview questions and probes (interview guides) with special focus: to comprehend the cancer care pathways; to understand stakeholders’ perception about the stages of cancer presentation; reasons for delayed presentation to seek the treatment; causes of non-compliance to prescribed treatment, and system level barriers to access cancer care services.

A female researcher with specialization/master’s in public health had conducted the interviews using mobile phone (amidst the second wave of Covid-19 in India). The interviewer was not related to any of the interviewed participants. Prior to the interview, the interviewer completely explained the aims and objectives of the study and need for the interview recording, and clarified the participants’ queries (if there were any) to seek an informed verbal consent. At the time of each interview, interviewer made sure that the participants were alone in the room. Each interview (for healthcare provider as well as patient) lasted for 35 to 40 minutes. All the interviews were audio-recorded and transcribed into word files.

For the analysis of qualitative data, open coding was done for the word transcripts and themes were generated to explore participants’ experiences related to existing cancer care services for the state of Telangana. The frequencies of codes were used for further analyses using Microsoft Excel 2016 edition. Wherever required, we have provided the supporting quotations (in the result section) from different participants to improve the transparency of the findings.

### Patient journey funnel development

We developed patient journey funnels for cancer care to highlight the levels of cancer care where leakages may occur. To develop this, a number of assumptions were made mostly based on the anecdotal evidence as the evidence-based findings were not available. We developed patient journey funnels for three common cancers together (oral, breast, and cervical) as well as separately for breast and cervical cancers. We developed the patient journey funnels for oral, breast, and cervical cancers because as a part of NPCDCS program the community level screening has been ongoing for these three cancers only since 2018 in the state of Telangana. We calculated patient leakages at various points and pathways across cancer care continuum such as (i) ‘Screened-positive to ‘diagnosis confirmation’; (ii) ‘Diagnostic confirmation’ to ‘treatment initiation’ and ‘treatment-completion’; (iii) ‘Screened-positive’ till ‘treatment-completion’; and (iv) ‘Confirmed-diagnosis’ till ‘treatment-completion’.

## Results

Table 1 describes the magnitude and corresponding workload (total number of visits required during pre- and post-diagnosis) for oral, breast, cervical, and lung cancers from 2022 to 2037.

**Table 1:** Estimated workload for the breast, cervical, oral, and lung cancer for the next 15 years (2022–37).

Year	CIR	New cases	Early stage	Late stage	Workload	Survival after 5^th^ year
Breast Cancer						
2022	50.42 (49.41, 51.44)	9500 (9310, 9692)	8168 (8005, 8333)	1332 (1305, 1359)	264852 (259543, 270207)	6433 (6304, 6563)
2027	54.53 (53.5, 55.58)	10515 (10316, 10717)	9041 (8870, 9214)	1474 (1446, 1503)	293136 (287590, 298771)	7120 (6985, 7256)
2032	58.98 (57.9, 60.06)	11538 (11328, 11750)	9921 (9739, 10103)	1618 (1588, 1647)	321674 (315794, 327575)	7813 (7670, 7956)
2037	63.79 (62.68, 64.91)	12585 (12366, 12806)	10820 (10632, 11011)	1764 (1734, 1795)	350836 (344748, 357013)	8521 (8373, 8671)
**Cervical Cancer**						
2022	12.36 (11.86, 12.87)	2328 (2235, 2425)	2158 (2071, 2248)	170 (163, 177)	91427 (87753, 95226)	1593 (1529, 1659)
2027	11.37 (10.9, 11.85)	2192 (2102, 2285)	2032 (1948, 2118)	160 (154, 167)	86094 (82533, 89726)	1500 (1438, 1564)
2032	10.46 (10.01, 10.93)	2047 (1958, 2138)	1897 (1815, 1982)	150 (143, 156)	80380 (76903, 83971)	1401 (1340, 1463)
2037	9.63 (9.2, 10.03)	1899 (1815, 1987)	1761 (1682, 1841)	139 (133, 145)	74588 (71276, 78016)	1300 (1242, 1359)
**Oral Cancer–Male**						
2022	25.88 (25.16, 26.61)	4934 (4797, 5073)	4514 (4389, 4642)	420 (408, 432)	192750 (187390, 198189)	2731 (2655, 2808)
2027	25.85 (25.14, 26.58)	5033 (4894, 5175)	4605 (4478, 4734)	428 (417, 440)	196618 (191189, 202140)	2786 (2709, 2864)
2032	25.83 (25.13, 26.55)	5087 (4949, 5229)	4654 (4528, 4784)	433 (421, 445)	198701 (193331, 204256)	2815 (2739, 2894)
2037	25.80 (25.10, 26.52)	5107 (4968, 5249)	4672 (4545, 4802)	435 (423, 447)	199490 (194061, 205040)	2826 (2750, 2905)
**Oral Cancer—Female**						
2022	4.97 (4.66, 5.30)	937 (878, 999)	857 (803, 914)	80 (75, 85)	36589 (34300, 39010)	518 (486, 553)
2027	4.87 (4.56, 5.19)	939 (879, 1001)	859 (804, 916)	80 (75, 85)	36664 (34347, 39093)	519 (487, 554)
2032	4.77 (4.46, 5.08)	932 (873, 994)	853 (798, 909)	79 (74, 85)	36425 (34085, 38824)	516 (483, 550)
2037	4.67 (4.37, 4.98)	921 (862, 983)	842 (789, 899)	78 (73, 84)	35968 (33679, 38380)	510 (477, 544)
**Lung Cancer—Male**						
2022	13.30 (12.79, 13.83)	2536 (2439, 2637)	1291 (1241, 1342)	1245 (1197, 1294)	76349 (73423, 79393)	254 (245, 264)
2027	13.10 (12.60, 13.62)	2551 (2453, 2652)	1299 (1249, 1350)	1252 (1204, 1302)	76797 (73857, 79836)	256 (246, 266)
2032	12.91 (12.41, 13.42)	2542 (2444, 2643)	1294 (1244, 1346)	1248 (1200, 1297)	76530 (73588, 79577)	255 (245, 265)
2037	12.71 (12.22, 13.22)	2516 (2419, 2617)	1281 (1231, 1332)	1235 (1187, 1284)	75764 (72822, 78781)	252 (243, 262)
**Lung Cancer- Female**						
2022	5.82 (5.48, 6.18)	1097 (1033, 1164)	559 (526, 593)	539 (507, 572)	33036 (31089, 35060)	110 (104, 117)
2027	5.80 (5.46, 6.15)	1118 (1053, 1186)	569 (536, 604)	549 (517, 582)	33656 (31699, 35705)	112 (106, 119)
2032	5.77 (5.44, 6.12)	1129 (1064, 1197)	575 (542, 610)	554 (522, 588)	33995 (32045, 36050)	113 (107, 120)
2037	5.75 (5.41, 6.09)	1133 (1067, 1201)	577 (543, 612)	556 (524, 590)	34127 (32137, 36176)	114 (107, 121)

CIR, crude incidence rate per 100000 individuals.

Estimated *female* populations for the years 2022, 2027, 2032, 2037 were 18842000, 19282000, 19564000, and 19729000, respectively; Estimated male populations for the years 2022, 2027, 2032, 2037 were 19066000, 19468000, 19694000, and, 19792000, respectively [9].

Proportion of breast cancer cases: Early stage = 85.98%, late stage = 14.02%.

Proportion of cervical cancer cases: Early stage = 92.69%, late stage = 7.31%.

Proportion of oral cancer cases: Early stage = 91.49%, late stage = 8.51%.

Proportion of lung cancer cases: Early stage = 50.91%, late stage = 49.09%.

A 5-year survival for breast cancer: Early stage = 0.7632, late stage = 0.1491.

A 5-year survival for cervical cancer; early stage = 0.7318; late stage = 0.0816.

A 5-year survival for oral cancer: Early stage = 0.6017, late stage = 0.0349.

A 5-year survival for lung cancer: Early stage = 0.1003, late stage = 0.1003.

Workload was defined as average number of visits to tertiary centers per case for consultations with oncologists, diagnostic investigations, active treatment and follow-up. Number of visits for Breast Cancer: Early stage = 29; late stage = 21; Number of visits for Cervical Cancer: Early stage = 40; late stage = 30; Number of visits for Oral cancer: Early stage = 40; late stage = 29; Number of visits for Lung Cancer: Early stage = 36; late stage = 24.

Breast cancer among females: Approximately 8.1%, 17%, and 26.5% increase in the crude incidence rate of breast cancer is anticipated for the year 2027, 2032, and 2037, respectively, compared to the year 2022 (50.42 new cases per 100000 female population). This will translate into 10515, 11538, and 12585 new breast cancer cases in the year 2027, 2032, and 2037 compared to 9500 new cases in 2022. The healthcare sector should expect a 10.7%, 21.5%, and 32.5% higher workload for the year 2027, 2032, and 2037, respectively, compared to the number of visits (n = 264852) in the year 2022.

Cervical cancer: Approximately 8.0%, 15.3%, and 22.1% decrease in the crude incidence rate of cervical cancer is anticipated for the year 2027, 2032, and 2037, respectively, compared to the year 2022 (12.36 new cases per 100000 female population). This will translate into 2192, 2047, and 1899 new cervical cancer cases in the year 2027, 2032, and 2037, respectively, compared to 2328 new cervical cancer cases in the year 2022. The healthcare sector should expect a 5.8%, 12.1%, and 18.1% lower number of patients’ visit for the year 2027, 2032, and 2037, respectively, compared to the number of visits (n = 91427) in the year 2022.

Oral cancer among females: Approximately 2.1%, 4.1%, and 6.1% decrease in the crude incidence rate of oral cancer among females is anticipated for the year 2027, 2032, and 2037, respectively, compared to the year 2022 (4.97 new cases per 100000 female population). This will translate into 939, 932, and 921 new oral cancer cases in the year 2027, 2032, and 2037 compared to 937 new cases in the year 2022. The healthcare sector should expect a 0.2% higher and 0.5%, and 1.7% lower number of patients’ visit for the year 2027, 2032, and 2037, respectively, compared to the number of visits (n = 36589) in the year 2022.

Lung cancer among females: Approximately 0.5%, 0.9%, and 1.3% decrease in the crude incidence rate of lung cancer among females is anticipated for the year 2027, 2032, and 2037, respectively, compared to the year 2022 (5.82 new cases per 100000 female population). This will translate into 569, 575, and 577 new lung cancer cases in the year 2027, 2032, and 2037 compared to 559 new cases in the year 2022. The healthcare sector should expect a 1.9%, 2.9%, and 3.3% higher number of patients’ visit for the year 2027, 2032, and 2037, respectively, compared to the number of visits (n = 33036) in the year 2022.

Oral cancer among males: Approximately 0.1%, 0.2%, and 0.3% decrease in the crude incidence rate of oral cancer among males is anticipated for the year 2027, 2032, and 2037, respectively, compared to the year 2022 (25.88 new cases per 100000 male population). This will translate into 5033, 5087, and 5107 new oral cancer cases in the year 2027, 2032, and 2037 compared to 4934 new cases in the year 2022. The healthcare sector should expect a 2%, 3.1%, and 3.5% higher number of patients’ visit for the year 2027, 2032, and 2037, respectively, compared to the number of visits (n = 192750) in the year 2022.

Lung cancer among males: Approximately 1.5%, 3%, and 4.4% decrease in the crude incidence rate of lung cancer among males is anticipated for the year 2027, 2032, and 2037, respectively, compared to the year 2022 (13.30 new cases per 100000 male population). This will translate into 2551, 2542, and 2516 new lung cancer cases in the year 2027, 2032, and 2037 compared to 2536 new cases in the year 2022. The healthcare sector should expect a 0.6% higher, 0.2% higher, and 0.8% lower number of patients’ visit for the year 2027, 2032, and 2037, respectively, compared to the number of visits (n = 76349) in the year 2022.

Overall, the magnitude of the disease defined as the total number of cases (new plus existing) will be 137%, 242%, and 330% higher for the year 2027, 2032, and 2037, respectively, compared to the year 2022 (n = 111942 total cancer cases) (S1 Table).

Table 2 describes the estimated chemotherapy, radiotherapy and surgery requirements for new cases of cancers from the year 2022 to 2037 under different scenarios. Across three assumed scenarios, the requirement of chemotherapy, radiotherapy as well as number of surgeries per year will be 13%, 28%, and 44.7% higher for the year 2027, 2032, and 2037, respectively, compared to the year 2022 due to increase in the number of new cancer cases. This would translate into:

For scenario 1 (55% newer cancer cases requiring chemotherapy and/or radiotherapy and remaining (45%) cases requiring surgeries): 512, 579, 654 chemo cycles per day in the year 2027, 2032, and 2037, respectively compared to 452 chemo cycles per day in the year 2022; 46, 52, and 59 newer LINACs for the year 2027, 2032, and 2037, respectively, compared to 41 newer LINACs in the year 2022; and 2122, 2400, and 2715 surgeries per month for the year 2027, 2032, and 2037, respectively, compared to 1876 surgeries per month in the year 2022.

For scenario 2 (60% newer cancer cases requiring chemotherapy and/or radiotherapy and remaining (40%) cases requiring surgeries): 558, 631, 714 chemo cycles per day in the year 2027, 2032, and 2037, respectively compared to 493 chemo cycles per day in the year 2022; 50, 57, and 64 newer LINACs for the year 2027, 2032, and 2037, respectively, compared to 45 newer LINACs in the year 2022; and 1886, 2133, and 2413 surgeries per month for the year 2027, 2032, and 2037, respectively, compared to 1667 surgeries per month in the year 2022.

For scenario 3 (65% newer cancer cases requiring chemotherapy and/or radiotherapy and remaining (35%) cases requiring surgeries): 605, 684, 773 chemo cycles per day in the year 2027, 2032, and 2037, respectively compared to 535 chemo cycles per day in the year 2022; 54, 62, and 70 newer LINACs for the year 2027, 2032, and 2037, respectively, compared to 48 newer LINACs in the year 2022; and 1650, 1867, and 2111 surgeries per month for the year 2027, 2032, and 2037, respectively, compared to 1459 surgeries per month in the year 2022.

**Table 2:** Estimated chemotherapy, radiotherapy, and surgery requirement in Telangana from 2022 to 2037.

Year	New cases	Estimated chemotherapy required		Estimated radiotherapy required		Estimated cancer surgeries required	
		New cases requiring chemotherapy	Chemo cycles/day	New cases requiring RT	LINACs require	New cases requiring surgery	Surgeries/ month
		55% of new cancer cases requiring chemo/radiotherapy*				45% of new cancer cases requiring surgery*	
2022	50024 (49318, 50463)	27513 (27125, 27755)	452 (446, 456)	27513 (27125, 27755)	41 (40, 41)	22511 (22193, 22708)	1876 (1849, 1892)
2027	56581 (55878, 57048)	31119 (30733, 31376)	512 (505, 516)	31119 (30733, 31376)	46 (46, 47)	25461 (25145, 25671)	2122 (2095, 2139)
2032	63998 (63504, 64497)	35199 (34927, 35473)	579 (574, 583)	35199 (34927, 35473)	52 (52, 53)	28799 (28577, 29024)	2400 (2381, 2419)
2037^**a**^	72388 (71861, 72916)	39813 (39524, 40104)	654 (650, 659)	39813 (39524, 40104)	59 (58, 59)	32575 (32337, 32812)	2715 (2695, 2734)
		**60% of new cancer cases requiring chemo/radiotherapy**				**40% of new cancer cases requiring surgery**	
2022	50024 (49318, 50463)	30015 (29591, 30278)	493 (486, 498)	30015 (29591, 30278)	45 (44, 45)	20010 (19727, 20185)	1667 (1644, 1237)
2027	56581 (55878, 57048)	33948 (33527, 34229)	558 (551, 563)	33948 (33527, 34229)	50 (50, 51)	22632 (22351, 22819)	1886 (1863, 1902)
2032	63998 (63504, 64497)	38399 (38102, 38698)	631 (626,636)	38399 (38102, 38698)	57 (56, 57)	25599 (25401, 25799)	2133 (2117, 2150)
2037	72388 (71861, 72916)	43433 (43117, 43750)	714 (709, 719)	43433 (43117, 43750)	64* (64, 65)	28955 (28744, 29166)	2413 (2395, 2431)
		**65% of new cancer cases requiring chemo/radiotherapy**				**35% of new cancer cases requiring surgery**	
2022	50024 (49318, 50463)	32516 (32057, 32801)	535 (527, 539)	32516 (32057, 32801)	48 (48, 49)	17509 (17261, 17662)	1459 (1438, 1472)
2027	56581 (55878, 57048)	36778 (36320, 37081)	605 (597, 610)	36778 (36320, 37081)	54 (54, 55)	19803 (19557, 19967)	1650 (1630, 1664)
2032	63998 (63504, 64497)	41599 (41277, 41923)	684 (679, 689)	41599 (41277, 41923)	62 (61, 62)	22399 (22226, 22574)	1867 (1852, 1881)
2037	72388 (71861, 72916)	47052 (46710, 47396)	773 (768, 779)	47052 (46710, 47396)	70 (69, 70)	25336 (25151, 25521)	2111 (2096, 2127)

LINACs, linear accelerators; RT, radiotherapy.

Chemotherapy requirement calculated based on six cycles per patient assumed; RT services requirement calculated considering 22 RT sessions per patient and 15000 RT sessions per machine assumed.

^**a**^For the year 2037, 55% of new cancer cases (i.e 39813) requiring chemo/radiotherapy and 45% of new cancer cases (i.e. 32575) requiring surgeries would translate into 654 extra chemotherapy cycles per day, 59 new linear accelerators to deliver radiotherapy and 2715 extra surgeries for cancer per month to cater the needs of new cancer case.

Table 3 describes the socio-demographic and clinical profile of cancer patients. Out of 17 cancer patients interviewed: 10 were males; 12 were married; 11 were educated up to senior secondary; 9 were from Telangana and 7 (41.2%) were from neighbouring states (Andhra Pradesh and Karnataka); 10 patients were residing in urban areas; and 15 patients reported to have some insurance. The neoplasm sites for the interviewed patients include: oral- (n = 6), breast (n = 3), lung (n = 3), gastrointestinal (n = 3), and cervix-uteri cancer (n = 1). 10 cancer patients were either in the third or fourth stage of cancer and 2 patients were unaware about their cancer staging. For 3 patients, cancer treatment was not initiated; whereas 14 patients were undergoing some form cancer treatment including chemotherapy and/or radiotherapy.

**Table 3:** Socio-demographic and clinical profile of patients (n = 17).

Variables	Categories	n (%)
Sex, n(%)	Male	10 (58.8)
	Female	7 (41.2)
Education, n(%)	Up to senior secondary	11 (64.7)
	Graduation & above	6 (35.3)
Home state, n(%)	Telangana	9 (52.9)
	Andhra Pradesh	6 (35.3)
	Bihar	1 (5.9)
	Karnataka	1 (5.9)
Marital status, n(%)	Married	12 (70.6)
	Widowed	3 (17.6)
	Divorced	1 (5.9)
	Never Married	1 (5.9)
Place of Habitual residence, n(%)	Urban	10 (58.8)
	Rural	7 (41.2)
Insurance status, n(%)	Government sponsored insurance	8 (47.1)
	Personal medical insurance	7 (41.2)
	No insurance	2 (11.7)
Neoplasm site, n(%)	Oral	6 (35.3)
	Breast	3 (17.4)
	Lung	3 (17.4)
	Gastrointestinal	3 (17.4)
	Cervix-uteri	2 (11.8)
Stage of cancer, n(%)	Fourth	8 (47.1)
	Third	2 (11.8)
	Second	1 (5.9)
	First	4 (23.5)
	Patient and/or caregiver unaware of cancer staging	2 (11.8)
Status of current treatment, n(%)	Radiotherapy only	6 (35.3)
	Chemotherapy only	5 (29.4)
	Chemo and radiotherapy	1 (5.9)
	Follow-up care	2 (11.8)
	Not initiated	3 (17.6)
Out of pocket expenses for treatment, n(%)	Yes	14 (82.4)
	No	3 (17.6)
Reference to tertiary care center, n(%)	Healthcare provider	8 (47.1)
	Extended family and /or friends	8 (47.1)
	Self	1 (5.9)

Table 4 describes the stakeholders’ perception about the stages of cancer presentation, reasons for delayed presentation, causes of non-compliance, and system level barriers to access cancer care services.

**Table 4:** Stages of cancer presentation, reasons for delayed presentation, causes of non-compliance, and system level barriers: Stakeholders perceptions.

Major cancers in Telangana and stage at presentation to oncologist (n = 25)			
Site of Cancer	Stage of Presentation	% of patients	Sector
Oral	III	40	Public
	II-III	70–80	Private
	III	50	Private-charitable
Breast	III	30	Public
	II-III	70	Private
	III-IV	60	Private-charitable
Cervix-uterine	III	30–40	Public
	II-III	80	Private
	III	50	Private-charitable
Lung	IV	40	Public
	II-IV	70	Private
	IV	90	Private-charitable
**Reasons enumerated by providers for delayed presentation (n = 25)**			n (%)
Lack of awareness of condition & Services			21 (84)
Stigma associated with diagnosis			19 (76)
Financial constraints			15 (60)
Rural background			12 (48)
Absence of family support/ caregiver to accompany			9 (36)
Preference to TCAM			5 (20)
**Causes for non-compliance to recommended therapy or referral advice (n = 25)**			n (%)
Psychological / Attitudinal barriers			15 (60)
Long distance–travel to higher centers			12 (48)
Myths and misconceptions			4 (16)
Accommodation concerns			2 (8)
**System Level Barriers (n = 25)**			n (%)
Lack of systematic / organized screening			15 (60)
Effects of pandemic on service delivery			13 (52)
Inadequate cancer care centers–infrastructure			13 (52)
Shortage of trained manpower			11 (44)
Limited supportive care for therapeutic			6 (24)
Referral pathways–linkages			6 (24)
Non-availability of essential drugs			4 (16)
Absence of standardized guidelines / protocols			3 (12)
Absence of requisite equipment			3 (12)

TCAM, traditional, complementary & Alternative medicine.

### Delayed presentation

#### Delayed presentation—Lack of awareness and determinants of health seeking behavior

Presentation to a healthcare facility in advanced stages was the common barrier perceived by health care providers at all levels of the health system in both public and private sectors.

84% of the healthcare providers mentioned lack of awareness as the main reason for delayed presentation. Inadequate knowledge on risk factors and screening measures such as procedure for self-examination; negligence of early symptoms and preference of local quacks to allopathic practitioners were the common patient-related barriers encountered by primary healthcare providers. Inadequate knowledge about the condition and available health care services was evident across patient-groups, with women and those from rural backgrounds being disproportionately affected.

*“We did not know earlier, approached several centers over the period of one year, used herbal medicines, have also been to Shivamogga in Karnataka”–[Patient: 17, Stomach Cancer]*

*I never knew about cancer*, *until I was diagnosed*. *I thought lump underneath breast was normal for pregnant and lactating women and believed cancer was a result of straining my voice at workplace–[Patient*: *1*, *Breast cancer*]

Educational status was associated with awareness on cancers, wherein none of the patients who were educated up to the senior secondary was aware of cancer as against 83.3% of patients among those who were graduates or more educated. The median duration from the initial identification of symptoms to receiving diagnosis was greater amongst patients with lower educational attainment and those with less awareness on cancer compared to those counterparts with higher educational status and greater awareness.

Medical officers both at primary and secondary levels of the state’s health system were concerned that social determinants such as literacy status, gender, religion, economic status and location of regular habitation (rural/urban/tribal) have a synergistic effect on individual health seeking behavior in general, and cancer prevention, in particular. The association of socioeconomic status with age at marriage, menstrual hygiene and sanitation and in turn, their role in infection-related cancers were highlighted by physicians and nurses at the secondary level of the public health system.

#### Delayed presentation—Stigma and misconception

Stakeholders at the community level emphasized that stigma was the prime reason responsible for the late presentation of cancer patients at health centers as villagers have a taboo and apprehension that revealing their diagnosis may lead to them being ‘labelled’ by the community which results in them facing hardships due to societal norms.

#### Delayed presentation—Financial barriers

While all the providers at tertiary level perceived financial constraints as a cause for delayed presentation, only 28.6% primary healthcare personnel had this perception. Also, 90% providers in the older age cohort perceived financial barriers delay healthcare-seeking as against 40% providers in the younger age cohort. Although diagnostic investigations and treatment would be provided without any user-fee in the public health facilities, 60% stakeholders contended that indirect costs in terms of loss of wages and productive work hours is a constraint for patients from families below the poverty line, more so if patient or primary caregiver is the sole earning member. Moreover, direct non-medical costs for transportation and accommodation ought to be borne by the patient or his family and affordability is a fundamental challenge for those from peripheral areas, as cancer treatment lasts for longer duration.

While 60% patients who had knowledge on cancer were not covered under any insurance scheme, 66.7% of patients who were not aware of the condition had govt. sponsored insurance scheme. 47.1% of the 17 cancer patients interviewed reported receiving treatment under the state-sponsored insurance scheme. As elucidated by providers, direct non-medical costs such as expenses for travel and accommodation were the major concerns of patients and their families. Owing to the unanticipated diagnosis, patients resorted to distress financing for funding current treatment. Out-of-pocket expenditure varied from 40% to 70%, due to limited coverage of cancer-related therapies under both state-sponsored and private medical insurance schemes.

#### Delayed presentation–Place of habitation and absence of support

While 85.7% of seven tertiary level healthcare providers opined that rural background is a determinant of delayed presentation, only 40% and 14.3% of providers at the secondary and primary levels, respectively, had the same concern. All the private sector healthcare practitioners perceived rural background as a barrier for early presentation compared to 35% of those serving in the public sector. Caregivers from rural backgrounds despite poor literacy had extreme difficulty navigating patients to the tertiary cancer centers aggregated in urban areas. Loss of employment and livelihood for the entire duration of treatment was reiterated by caregivers. Caregiving resulted in role-conflicts and primary caregivers also experienced emotional trauma as some of them did not reveal the exact diagnosis to either the patient or other members of the family.

### Non-compliance to recommended therapy

Oncologists mentioned that patients’ perception of cancer treatments and subsequent side-effects such as loss of hair during chemotherapy, propel them to default the initiated therapy and search for alternatives under the assumption that the recommended treatment is ineffective. Perceived barriers behind treatment defaulting include beliefs that cancer is incurable, or radiotherapy would result in burns or death of the patient and that cancer patients receiving treatment should consume only plant-based diet.

*“Once patients return home and after the first cycle*, *they feel they are completely cured and don’t return for the next cycle on time*, *until the disease progresses and return when they start experiencing discomfort/difficulty*.”–Provider 3

“Why did this come to me? No one in our family has this problem. Then why did this happen to me?”—[Patient: 5, Breast Cancer]

60% healthcare providers expressed psychological concerns such as anxiety as a cause for non-compliance. Social circumstances such as absence of family support and /or caregiver to accompany the patient and distant location of diagnostic and treatment centers were considered by providers as the causes for non-compliance to referral advice and recommended therapy. While 20% of oncologists perceived supportive care as a barrier, 66.7% of nurses along with both the palliative care physicians and the stakeholder from community-based NGO perceived it as a barrier for patients.

While cancer patients who experienced accommodation/shelter barriers had an average duration of 4.5 months from noticing symptoms to being diagnosed with cancer, this duration was only 1.5 months for those without such concerns. In comparison to patients without transportation barriers, those with such challenges had a longer interval between first identification of symptoms and receiving the confirmatory diagnosis.

### System-level barriers

System-induced barriers mainly consisted of issues concerning infrastructure and workforce essential for service-delivery (including screening), in addition to ill-effects of the pandemic. Lack of systematic/ organized screening programs, effect of the COVID pandemic and inadequate infrastructure were the three most important barriers identified by health care providers (Table 3). Health care providers at the primary level identified challenges in the implementation of the NPCDCS program, which impacts cancer care services in Telangana: (i) ANMs in few districts not trained in screening for cervical cancer; (ii) Risk of missing cases at the primary level; (iii) Complex reporting formats; (iv) Multiple roles assigned to ANMs under the existing health care programmes (reproductive health programs and NPCDCS) in the scheduled work hours and (iv) Reduced workforce efficiency. At the primary level, screening activities have had been initiated in the state but ANMs only in certain districts were trained in early detection of all the three common cancers–breast, cervical and oral. Although, ANMs were conducting oral visual examination and clinical breast examination, training in VIA-based (Visual Inspection with Acetic acid) screening for early detection of cervical cancer hadn’t been completed in some of the districts.

Absence of histopathology labs, diagnostic equipment and chemo drugs for onco-specific treatment at the district level, including medical colleges in the public sector was identified as the principal barrier by providers at the secondary level. At the tertiary level, absence of designated areas for mixing of chemo-drugs and deficit of protective gear required during the process were some of the challenges faced by nursing personnel in the public sector. Despite the presence of shift-duties, due to extensive patient-volumes at the tertiary cancer centers in the public sector, provision of quality care was a perceived barrier. In addition to patient-care, administration of chemotherapy and diet-maintenance, nurses were engaged in documentation and ‘clerical work’, exacerbating the problem.

*Patient mainly expect us to listen to their problems*. *But we can’t spend enough time with each of them*.*”–[Provider*: *12*]

### System level barriers–Patient care pathways

Absence or ill-defined standardized pathways for cancer management (aimed to ensure timely diagnosis followed by prompt and appropriate treatment) was another important barrier highlighted by healthcare providers. Physicians at the primary healthcare level opined that referring to secondary level health centers is of limited use as there is a shortage of specialists and equipment for confirmatory diagnosis and staging. At the secondary level, medical officers and civil assistant surgeons highlighted the need for standard protocols for providers to ensure appropriate referral of screen-positives and suspected cases.

**Fig 1:**
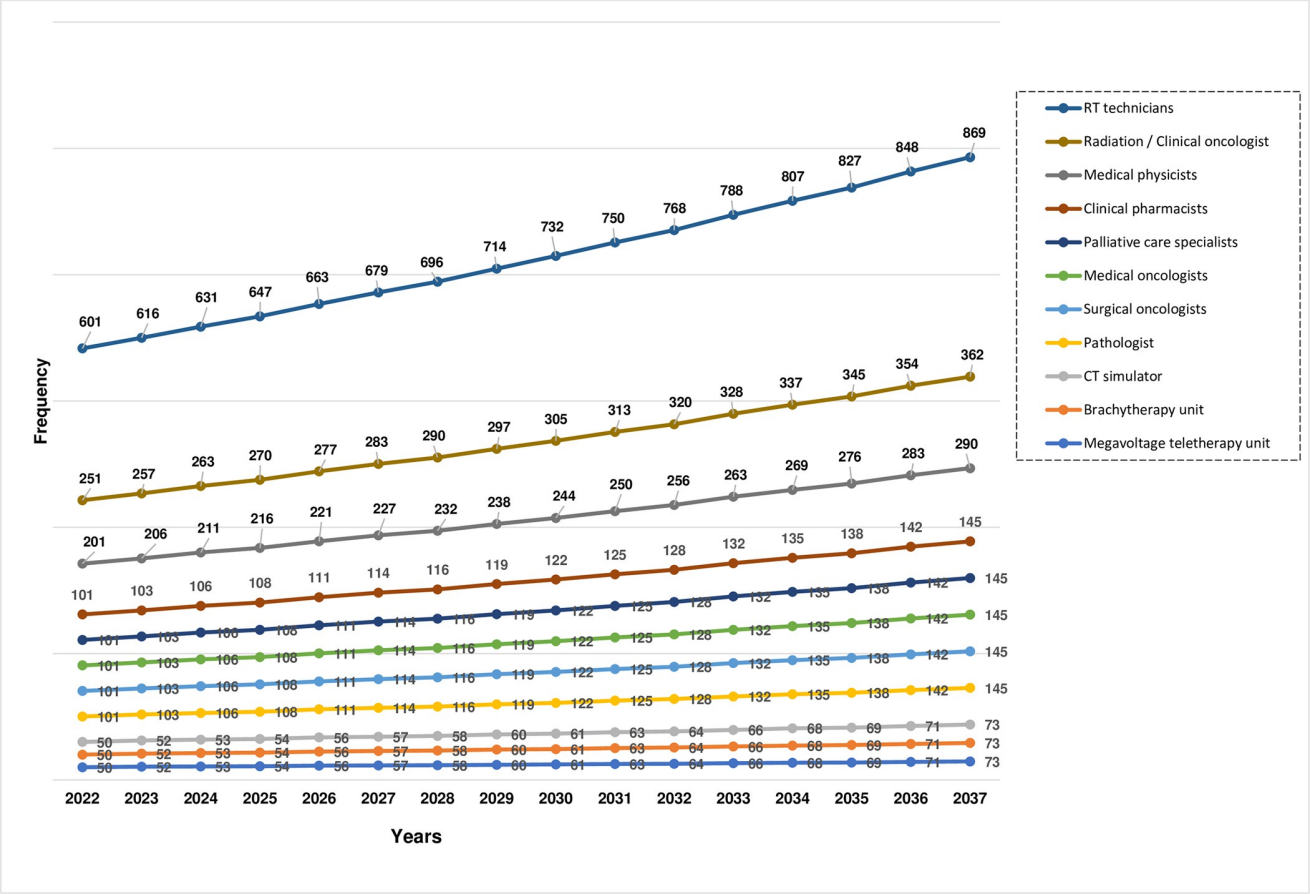
Human resources and equipment requirement for new cancer cases from 2022–2037. RT, radiotherapy; CT, computed tomography. Estimated new cancer cases for the year 2022, 2023, 2024, 2025, 2026, 2027, 2028, 2029, 2030, 2031, 2032, 2033, 2034, 2035, 2036, and 2037 are 50024, 51272, 52550, 53861, 55204, 56581, 57992, 59438, 60921, 62440, 63998, 65594, 67230, 68907, 70626, and 72388, respectively. The recommended norms per 1000 new cancer cases [12] for Megavoltage teletherapy unit, Brachytherapy unit, CT simulator, Pathologist, Surgical oncologists, Medical oncologists, Palliative care specialists, Clinical pharmacists, Medical physicists, Radiation / Clinical oncologist, and Radiotherapy technicians are 1, 1, 1, 2, 2, 2, 2, 2, 4, 5, and 12, respectively.

The required number of human resources and equipment for new cancer cases from year 2022 to 2037 are summarized in Fig 1. There is a steady increase in the requirement of human resources and equipment per year with rising number of new cancer cases. The requirement of human resources (such as Pathologist, Surgical oncologists, Medical oncologists, Palliative care specialists, Clinical pharmacists, Medical physicists, Radiation / Clinical oncologist, and Radiotherapy technicians) and equipment (Megavoltage teletherapy unit, Brachytherapy unit, CT simulator) will be approximately 13%, 28%, 44.7% higher in the year 2027, 2032, and 2037, respectively, compared the year 2022.

**Fig 2:**
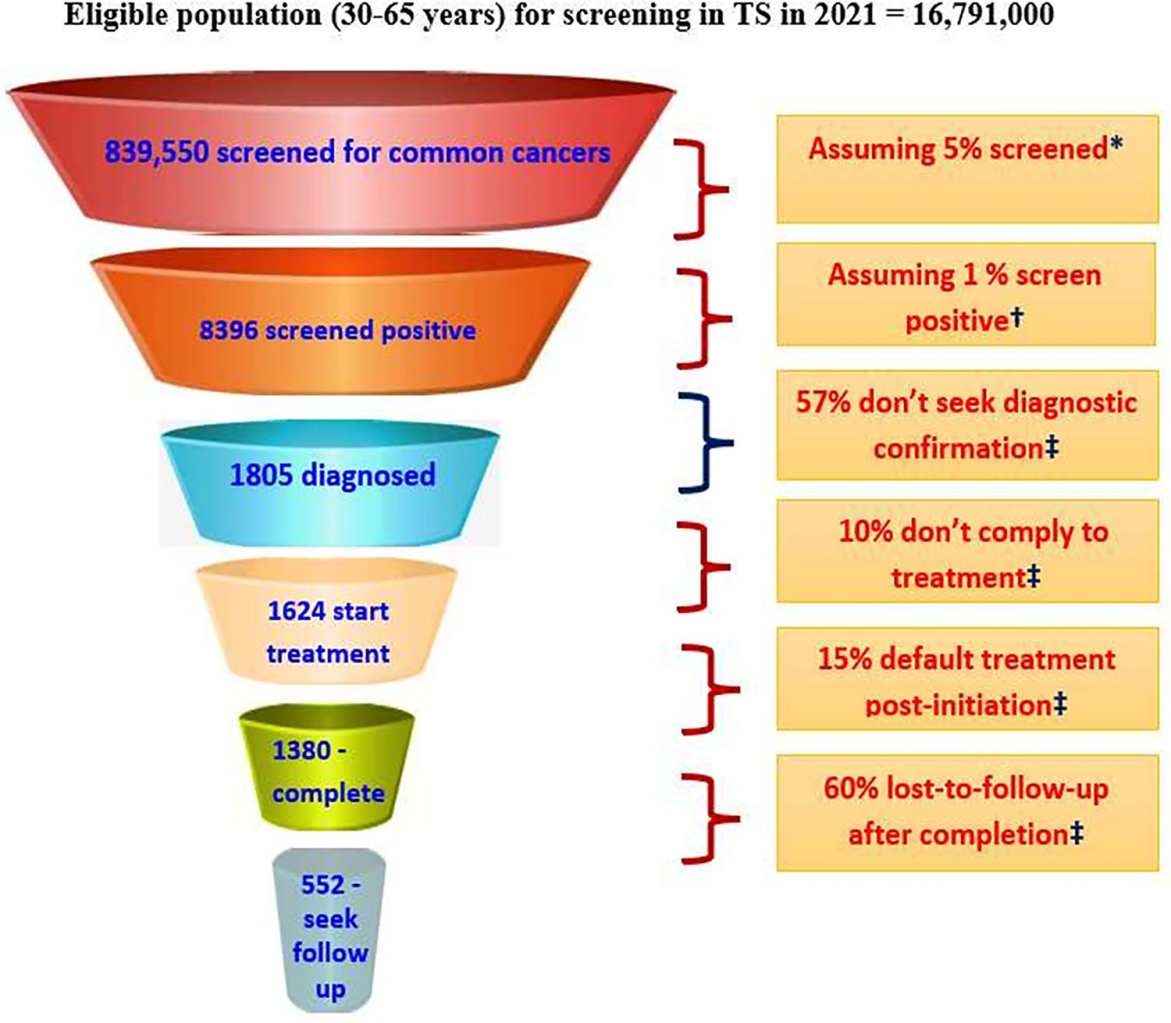
Patient leakage at various stages in the continuum of cancer care for breast, cervical and oral cancer together. *Extrapolated from percentage screened amongst 30–49 years age-group according to NFHS-5. †Based on age-truncated case detection rate of cancers (of all sites) in India [13]. ‡3610 (43%) seek diagnostic confirmation, assuming 50% of these to be cases. ‡Based on interviews with oncologists. (a) Total patient loss (from ‘screened-positive’ till ‘treatment-completion’ step) = [8396–552]/[8396] = 93.4%; (b) Assuming all ‘screened-positive’ seek ‘diagnostic-confirmation’ & 50% of which test positive, patient loss = [(8396/2) -552] / [8396/2] = 87.0%; (c) From ‘confirmed-diagnosis’ till ‘treatment-completion’ step, patient loss = (1805–552) /1805 = 69.4%.

Experts reported leakage of patients from screened positive till treatment completion in Fig 2. Major leakage of patient was highlighted between ‘screened-positive’ to ‘diagnosis confirmation’ step. Overall from ‘screened-positive’ till ‘treatment completion’, patient leakage varied from ~70% to 90%. The findings were relatively same when we developed patient leakage funnels separately for cervical and breast cancers (S1A and S1B Fig).

## Discussion

We conducted a situational analysis of cancer care activities for the state of Telangana to identify potential public health value additions and complement the cancer care initiatives of the State Government. We found multiple challenges in cancer care activities that need attention to influence cancer prevention in the state of Telangana. These will be: (i) 13%, 28%, 44.7% increase in the number of new cancer cases and 137%, 242%, 330% increase in the number people living with cancer for the year 2027, 2032, and 2037, respectively, compared to the year 2022; (ii) Proportionate increase (i.e. 13%, 28%, and 44.7%) in the workload on the healthcare system (for example number of patients’ visits, chemotherapy sessions /radiotherapy sessions, number of surgeries per day) is also anticipated for the year 2027, 2032, and 2037, respectively, compared to the year 2022; (iii) 13%, 28%, and 44.7% higher annual requirement of human and equipment for the year 2027, 2032, and 2037, respectively, compared to the year 2022 to cater the increasing workload; (iv) Suboptimal treatment seeking behavior among patients (delayed presentation to hospital for cancer care) accompanied with non-compliance to treatment due to individual as well as system level barriers; (v) Major patient leakage of 70% to 90% from ‘screening’ till ‘treatment completion’ for common cancers. To our knowledge, this is the first study from India, which using mixed method has assessed the human and equipment requirement for the rising burden of cancer and highlighted the individual- and system-level barriers for major patient leakage across cancer care continuum.

### Delayed access to healthcare system

This study has identified several public health additions that should be addressed to further catalyze the prevention and control of cancers in Telangana. Stakeholders’ repeatedly observed the delayed presentation of cancer patients, which is the main reasons for the poor survival of cancer patients in India compared to their European counterparts. Most of the stakeholders reported lack of awareness or misconception about cancer, its risk factors followed by financial constraints. This finding is consistent with the results reported from Orissa (one of the neighboring state of Telangana) where researchers quantified the delays in care-seeking for signs and symptoms related to cancer [14]. They found that the first step in the cancer pathway-to-care was sharing symptoms or signs with family members and friends, and on average, took 271 days before steps toward diagnosis were taken [14]. Lack of knowledge, fear, and stigma related to cancer were highlighted as the key factors influencing this delay [14]. The cancer-literacy plays a key role in cancer outcomes and the government should consider investing significantly in cancer prevention education. Our findings also highlight a need: (i) To identify effective measures to promote cancer literacy across all stakeholders; (ii) To monitor and evaluate these strategies to achieve optimum benefit; (iii) To gain the in-depth understanding of socio-cultural factor such as myths, misconception etc. and other barriers to access cancer care services.

Stakeholders also mentioned about the financial constraint as one of the major deterrents for early access to healthcare facility; and therefore cancer prevention efforts should ensure access to affordable treatment. Cancer patients in India incur a heavy out-of-pocket expenditures [15,16]. Several central as well as state insurance schemes exist for cancer patients in Telangana to cover different aspects of cancer care. Some of the schemes are: Rashtriya Aarogya Nidhi Health Minister’s Cancer Patient Fund, The Employees’ State Insurance scheme, Telangana state Aarogyasri scheme, The Employees and Journalists Health Scheme of Telangana etc. that cover cancer care including specialized investigation as well as surgery, chemotherapy, radiotherapy, and palliative chemotherapy [17]. Government should facilitate the regular monitoring and evaluation of these schemes to ensure that these schemes cover all the components across cancer care continuum. The data from National Sample Survey- 75th round points to 70.3% of rural and 37.3% urban households in Telangana being covered under a government sponsored health insurance scheme. In case of hospitalized cancer patients, 83% were admitted at private hospitals from those hailing from rural Telangana as against 70% from those belonging to urban areas. Overall, 79% of hospitalized cancer patients in Telangana were admitted in private hospitals who might have incurred huge out-of-pocket expenses if had not been covered under any insurance scheme [18].

### Patient leakage across cancer care continuum

This study also highlighted the variable leakage of patients across cancer care continuum with a major leakage happening from ‘screening’ to ‘diagnosis-confirmation’ stage. The loss of patients across cancer care continuum could be explained by factors operating at three levels: a) Reduced generation and dissemination of scientific knowledge regarding interventions, practices, and services; b) Inadequately prepared healthcare system delivering inefficient services; and c) Poor demand and uptake of cancer services due to social, cultural and economic factors. Rigorous research across these three levels could identify needs and gaps in the existing cancer care system, which if addressed, could catalyze the cancer care services in the state of Telangana. As suggested previously [19], WHO’s six building blocks of the health system framework consisting of: information, medical products and technologies, service delivery, health workforce, financing, and leadership and governance can be used to identify research needs to promote generation and dissemination of scientific knowledge regarding interventions, practices and services and strengthen healthcare system to deliver inefficient services. Additionally, poor demand and uptake of cancer services due to social, cultural and economic factors can be mitigated by tackling context-specific culturally relevant cancer prevention education.

The government of Telangana has been showing a strong commitment and taking major initiatives to strengthen the cancer care services across the cancer care continuum (S3 Table). Telangana government is attempting to develop a decentralized cancer care system comprising stakeholders from public, public-private partnership, and non-governmental institutes to provide equitable, accessible, affordable, safe, and effective cancer care services [20]. The government is planning to have an apex center in Hyderabad (Level-1 cancer care center with facilities for advanced diagnostics, treatment, research, and education) in addition to existing MNJ Cancer Hospital, Nizam’s Institute of Medical sciences, and several private hospitals in Hyderabad. Medical colleges at Adilabad, Nizamabad, Mahbubnagar and Warangal are being strengthened as Level-2 centres to provide diagnostic and treatment services in all modalities such as surgical care, radiotherapy, and chemotherapy. Diagnostic hubs at the level of district (Level-3 facilities) to provide definitive diagnostics, day-care and palliative therapy. The government is actively promoting the training of ANMs at sub-center (SC) and medical officer at PHC, and CHC, to efficiently utilize the extensive network of SCs, health wellness canters (HWCs), PHCs, and CHCs to create awareness and provide basic screening services to ensure early diagnosis and prevent future cases. Additionally, several initiatives (by governmental and non-governmental organizations) are being carried out to -raise cancer literacy, -address fear and stigma, -formulate, -disseminate, and -implement evidence-based guidelines for screening, diagnosis and treatment (including palliative care), -increase cancer care centers/institutes and -address shortages in healthcare facilities, medical resources, and healthcare professionals to ensure timely care-seeking and follow- up (S3 Table). These newer strategies should be accompanied with rigorous monitoring and evaluation tools to identify appropriate service delivery and financing models.

### Strengths and limitations

For in-depth interviews, we have considered cancer patients (with different organ involvement and were at different stages) and healthcare providers working at different levels of healthcare to understand the complete dimension of cancer care services. This activity also helped us to develop patient leakage funnel in the absence of scientific evidence to appreciate at what levels of care patient leakages occur due to sub-optimal efforts and therefore required extra efforts.

The findings highlighted in this paper should be considered in the light of limitations. First, the number of patients as well as healthcare providers interviewed were very small. However, we attempted to achieve the thematic saturation and tried to gain diverse information. The in-depth interviews helped us to highlight the issues in cancer care (at patient- as well as system-level) which need urgent attention to strengthen the cancer care services; Second, we could not find out the existing number of human resources and equipment in the state of Telangana due to limited access to public as well private healthcare institutes; therefore could not calculate the exact current deficit of these resources. However, interviews with different stakeholders working at different level of healthcare gave us the glimpse of limited availability of these resources across various sectors. Third, we were only able to conduct phone interviews due to the COVID-19 pandemic, which may have led to altered responses. However, the interviewers were adequately trained before the interviews to extract relevant information from the stakeholders. Fourth, we made several assumptions and used anecdotal evidence to calculate the expected burden for cancer in next 15 years as well as to highlight patient leakages in cancer care funnel. Although we were unable to calculate the exact numbers for projections as well as patient leakages, we have delineated a dual nature of problem in cancer care: rising number of cases accompanied by poor availability and/or utilization of healthcare system.

### Public health implications

Amidst globalization, many lower middle income countries (including India) are experiencing urbanization, dietary transition (from fruits and green leafy vegetables to calorie-rich and nutrient sparse fried food), and changes in environmental exposures which could increase the burden of overweight, obesity, and non-communicable diseases including cancer. Policymakers in India are struggling to identify optimal strategies to allocate limited resources for non-communicable diseases as these are already being utilized for communicable diseases and maternal and child health. This study very clearly highlighted the dual problems for cancer care: rising burden and delayed access to healthcare system, in a population which is experiencing epidemiological transition (due to aging and lifestyle changes). Additionally, we have highlighted the specific areas in the cancer care continuum which needs attention (for example more implementation research) to facilitate early diagnosis of cancer and thereby better prognosis. Improving the health of transitional population, for instance through cancer prevention and control will positively impact economic productivity in India. This will be particularly significant south Asians who tend to develop cancer at earlier ages (during their economic productive years).

## Conclusion

Telangana state has made commendable efforts towards improving the health profile of its citizens. However, there is ample scope for improvement in the delivery of comprehensive cancer care services in the state. This study has highlighted multiple challenges in the cancer care services: Rising burden of cancer cases; Increasing requirement of human and equipment to cater the rising cancer cases; Delayed access to healthcare services; Geographical disparity to access cancer care services; and Major patient leakage across cancer care continuum. To manage this public health issue, government should take appropriate measures to improve cancer-literacy at the community level as well as increase specialized human resources and necessary equipment. Further, implementation research focusing on the identified challenges/barriers is required to identify the optimum strategy to prevent cancer and promote the uptake of cancer care services.

## Supporting information

S1 Checklist COREQ (COnsolidated criteria for REporting Qualitative research) checklist. (DOCX)

S1 Fig A. Patient leakage at various stages in the continuum of breast cancer care. B. Patient Leakage at various stages in the continuum of cervical cancer care.
(PDF)

S1 Table Estimated magnitude of cancers in Telangana for next 15 years (2022 to 2037). (DOCX)

S2 Table Socio-demographic and designation of healthcare providers (n = 25). (PDF)

S3 Table Cancer care initiatives in the state of Telangana. (DOCX)

S1 Appendix The Telangana Cancer Control Study Group. (PDF)
