# Supplementary material for: Projected cancer burden, challenges, and barriers to cancer prevention and control activities in the state of Telangana
Source: PLoS One. 2023 Jul 14;18(7):e0278357. doi: 10.1371/journal.pone.0278357 (PMC10348541; doi:10.1371/journal.pone.0278357)
Supplement: S1 Checklist — (DOCX) [file pone.0278357.s001.docx]

**COREQ (COnsolidated criteria for REporting Qualitative research) Checklist**

| **Topic** | **Item no.** | **Questions / Descriptions** | **Reported on page no.** |
| --- | --- | --- | --- |
| **Domain 1: Research team and reflexivity** | | | |
| *Personal characteristics* | | | |
| Interviewer/facilitator | 1 | Which author/s conducted the interview or focus group? | Ms. Neha Reddy; Page no. 1 & 11 |
| Credentials | 2 | What were the researcher’s credentials? | Master’s in Public Health; Page no. 1 & 11 |
| Occupation | 3 | What was the occupation at the time of the study? | Research Assistant; Page no. 11 |
| Gender | 4 | Was the researcher male or female? | Female; Page no. 11 |
| Experience & Training | 5 | What experience or training did the researcher have? | Master's in Public Health; Page no. 11 |
| *Relationship with participants* | | | |
| Relationship established | 6 | Was a relationship established prior to study commencement? | Yes; Page no. 11 |
| Participant knowledge of the interviewer | 7 | What did the participants know about the researcher? e.g. personal goals, reasons for doing the research | Reasons for doing the research; Page no. 11 |
| Interviewer characteristics | 8 | What characteristics were reported about the inter viewer/facilitator?  e.g. Bias, assumptions, reasons and interests in the research topic | None |
| **Domain 2: Study design** | | | |
| *Theoretical framework* | | | |
| Methodological orientation and Theory | 9 | What methodological orientation was stated to underpin the study? | Descriptive Phenomenological Method; Page no. 11 |
| *Participant selection* | | | |
| Sampling | 10 | How were participants selected? | Purposive; Page no. 11 |
| Method of approach | 11 | How were participants approached? e.g. face-to-face, telephone, mail, email | Telephone; Page no. 11 |
| Sample size | 12 | How many participants were in the study? | 43; Page no. 11 |
| Non-participation | 13 | How many people refused to participate or dropped out? Reasons? | 5 participants refused to participate due to lack of time. |
| *Setting* | | | |
| Setting of data collection | 14 | Where was the data collected? e.g. home, clinic, workplace | Data was collected using telephone; The participants were in hospital at the time of interview. Page no 11 |
| Presence of non- participants | 15 | Was anyone else present besides the participants and researchers? | None |
| Description of sample | 16 | What are the important characteristics of the sample? e.g. demographic data, date | Socio-demographic features; Supplemental tables 2 & 3 |
| *Data collection* | | | |
| Interview guide | 17 | Were questions, prompts, guides provided by the authors? Was it pilot tested? | The prompts and guides were provided by the authors; However, they were not pilot tested. |
| Repeat interviews | 18 | Were repeat inter views carried out? If yes, how many? | No |
| Audio/visual recording | 19 | Did the research use audio or visual recording to collect the data? | Yes; Page no. 11 |
| Field Notes | 20 | Were field notes made during and/or after the interview or focus group? | Yes |
| Duration | 21 | What was the duration of the inter views or focus group? | 35 to 40 minutes; Page no. 12 |
| Data saturation | 22 | Was data saturation discussed? | No |
| Transcripts returned | 23 | Were transcripts returned to participants for comment and/or correction? | No |
| **Domain 3: Analyses and Findings** | | | |
| *Data Analyses* | | | |
| Number of data coders | 24 | How many data coders coded the data? | One |
| Description of the coding tree | 25 | Did authors provided a description of the coding tree? | No |
| Derivation of Themes | 26 | Were themes identified in advance or derived from the data? | Advance; Page No. 11 |
| Software | 27 | What software, if applicable, was used to manage the data? | Microsoft excel; Page no. 11 |
| Participant Checking | 28 | Did participants provide feedback on the findings? | No |
| Reporting | | | |
| Quotation presented | 29 | Were participants quotation presented to illustrate the themes/findings? Was each quotation identified? | Yes; Page no. 22 to 26 |
| Data and findings consistent | 30 | Was there consistency between the data presented and the findings? | Yes |
| Clarity of Major themes | 31 | Were major themes clearly presented in the findings? | Yes; Page no 20 to 25 |
| Clarity of Minor themes | 32 | Is there a description of diverse cases or discussion of minor themes? | No |

Developed from: Tong A, Sainsbury P, Craig J. Consolidated criteria for reporting qualitative research (COREQ): a 32-item checklist for interviews and focus groups. *International Journal for Quality in Health Care*. 2007. Volume 19, Number 6: pp. 349 – 357.
