# Supplementary material for: Projected cancer burden, challenges, and barriers to cancer prevention and control activities in the state of Telangana
Source: PLoS One. 2023 Jul 14;18(7):e0278357. doi: 10.1371/journal.pone.0278357 (PMC10348541; doi:10.1371/journal.pone.0278357)
Supplement: S1 Fig — A. Patient leakage at various stages in the continuum of breast cancer care. B. Patient Leakage at various stages in the continuum of cervical cancer care. (PDF) [file pone.0278357.s002.pdf]

S1A Fig. Patient leakage at various stages in the continuum of breast cancer care

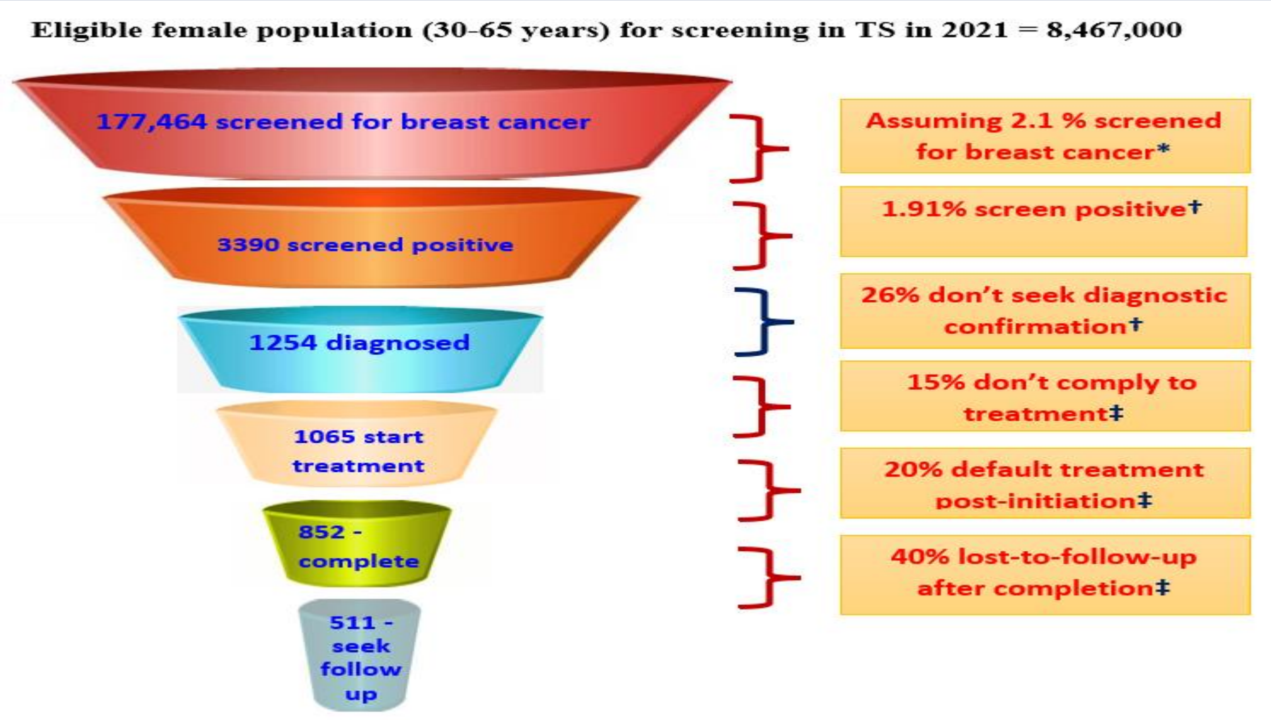

*****0.3% screened for breast cancer in the age group of 30-49 years (NFHS-5) and 177,464 women above 30 years screened during 2020-21 [1].

†Determinants of compliance to clinical breast examination [2]

‡Based on interviews with oncologists (considering both rural and urban scenarios)

(a) Total patient loss (from screened positive till treatment completion) = [3390-511]/[3390]= **84.9%**

(b) Assuming all screened positive seek diagnostic confirmation & 50% of which test positive, patient loss= [(3390/2)-511]/[3390/2]= **69.8%**

(c) From confirmed diagnosis till treatment completion, patient loss =(1805 – 552) /1805= **59.2%**

References

[1] Record of proceeding telangana 2021-2022 national health mission [internet]. 2021 [accessed on: 2021 aug 29].
[2] Kulkarni SV, Mishra GA, Dusane RR. Determinants of compliance to breast cancer screening and referral in low socio-economic regions of urban india. International journal of preventive medicine. 2019;10:84. doi:10.4103/ijpvm.IJPVM_335_17

S1B Figure. Patient Leakage at various stages in the continuum of cervical cancer care

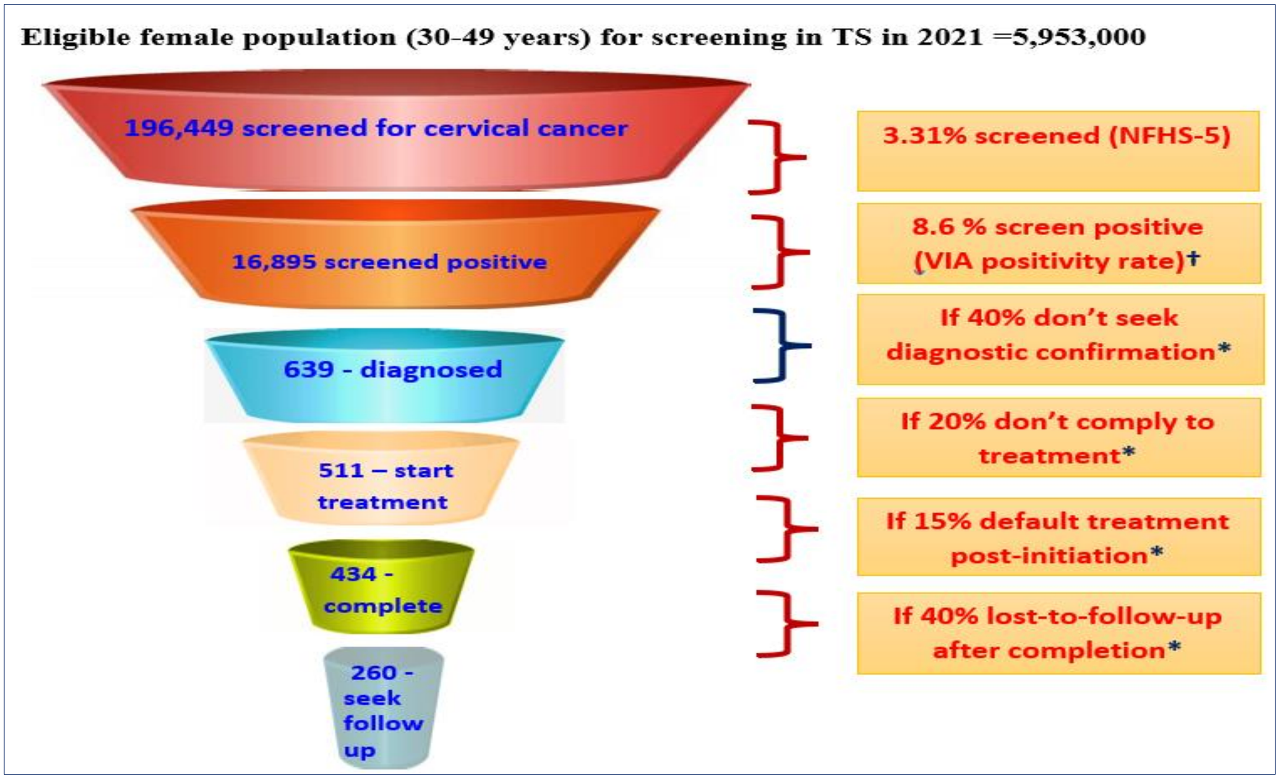

†If all screen -positives seek diagnostic confirmation, 6.3% of screen positives (0.54% of all screened) [1] would have invasive cervical cancer. Since, only 10,137 (60%) seek diagnostic confirmation, 639 diagnosed.

*****Based on interviews with specialists

(a) Total patient loss (from screened positive till treatment completion) = [16895-260]/[16895**]= 98.5%**

(b) Assuming all screened positive seek diagnostic confirmation & 6.3% of which test positive, patient loss= [(16895*0.063)-260]/[16895*0.063]= **75.6%**

(c) From confirmed diagnosis till treatment completion, patient loss =(1805 – 552) /1805= **59.3%**

References

[1] Poli UR, Bidinger PD, Gowrishankar S. Visual inspection with acetic acid (via) screening program: 7 years experience in early detection of cervical cancer and pre-cancers in rural south india. Indian Journal of Community Medicine 2015;40:203-7. doi:10.4103/0970-0218.158873
