# Supplementary material for: Projected cancer burden, challenges, and barriers to cancer prevention and control activities in the state of Telangana
Source: PLoS One. 2023 Jul 14;18(7):e0278357. doi: 10.1371/journal.pone.0278357 (PMC10348541; doi:10.1371/journal.pone.0278357)
Supplement: S1 Table — (DOCX) [file pone.0278357.s003.docx]

**S1 Table**. Estimated magnitude of cancers in Telangana for next 15 years (2022-2037)

| Year | Prevalent cases at the start of the year | Yearly incidence | Est. total population | New cases added during the year | Total cases in the year | Total cases surviving at the end of the year |
| --- | --- | --- | --- | --- | --- | --- |
| 2022 | 73858 | 96.93 | 37908000 | 36743 | 110601 | 99541 |
| 2023 | 99541 | 98.88 | 38090000 | 37662 | 137202 | 123482 |
| 2024 | 123482 | 100.87 | 38271000 | 38603 | 162085 | 145877 |
| 2025 | 145877 | 102.90 | 38454000 | 39568 | 185445 | 166900 |
| 2026 | 166900 | 104.97 | 38636000 | 40557 | 207458 | 186712 |
| 2027 | 186712 | 107.28 | 38750000 | 41571 | 228283 | 205455 |
| 2028 | 205455 | 109.64 | 38864000 | 42611 | 248066 | 223259 |
| 2029 | 223259 | 112.05 | 38979000 | 43676 | 266935 | 240242 |
| 2030 | 240242 | 114.52 | 39092000 | 44768 | 285009 | 256508 |
| 2031 | 256508 | 117.04 | 39207000 | 45887 | 302395 | 272156 |
| 2032 | 272156 | 119.81 | 39258000 | 47034 | 319190 | 287271 |
| 2033 | 287271 | 122.64 | 39311000 | 48210 | 335481 | 301933 |
| 2034 | 301933 | 125.54 | 39363000 | 49415 | 351348 | 316213 |
| 2035 | 316213 | 128.51 | 39415000 | 50651 | 366864 | 330178 |
| 2036 | 330178 | 131.54 | 39468000 | 51917 | 382094 | 343885 |
| 2037 | 343885 | 134.65 | 39521000 | 53215 | 397100 | 357390 |
