# Supplementary material for: Projected cancer burden, challenges, and barriers to cancer prevention and control activities in the state of Telangana
Source: PLoS One. 2023 Jul 14;18(7):e0278357. doi: 10.1371/journal.pone.0278357 (PMC10348541; doi:10.1371/journal.pone.0278357)
Supplement: S2 Table — (PDF) [file pone.0278357.s004.pdf]

S2 Table. Socio-demographic and designation of healthcare providers

Variables	Categories	n (%)
Sex	Male	13 (52)
	Female	12 (48)
Education	Secondary & below	2 (8)
	Graduation	12 (48)
	PG Diploma	2 (8)
	Post-graduation	9 (36)
Sector	Public	20 (80)
	Private	4 (16)
	NGO	1 (4)
Health System Level	Primary	7 (28)
	Secondary	10 (40)
	Tertiary	7 (28)
	Community-based NGOs	1 (4)
Designation	Oncologist	5 (20)
	Pulmonologist	1 (4)
	Civil Assistant Surgeons	2 (8)
	Medical Officers - District Hospital	1 (4)
	Medical Officers - CHC	1 (4)
	Medical Officers - PHC	5 (20)
	Palliative care officer - NCD	2 (8)
	Staff Nurses	2 (8)
	ANMs	3 (12)
	Mid-level Health Providers	1 (4)
	NCD - Programme Officer	1 (4)
	NGO secretary	1 (4)
