# Supplementary material for: Projected cancer burden, challenges, and barriers to cancer prevention and control activities in the state of Telangana
Source: PLoS One. 2023 Jul 14;18(7):e0278357. doi: 10.1371/journal.pone.0278357 (PMC10348541; doi:10.1371/journal.pone.0278357)
Supplement: S3 Table — (DOCX) [file pone.0278357.s005.docx]

**S3 Table**: Cancer care initiatives in the state of Telangana

| **Level of Prevention** | **Initiatives** |
| --- | --- |
| Primary (risk factor assessment and high risk group identification) | a) Use of ‘ASHA-disease profile’ app to capture information about risk factors and symptoms of various diseases (including cancer) for all individuals in the State;  b) Set-up of district level coordination committees and enforcement squads in 27 districts;  c) Tobacco cessation services in 177 SCs/PHCs across 20 districts (as a part of implementation of National Tobacco Control Programme);  d) Health camps to provide information and guidance on available cancer care services as part of Aarogyasri scheme  e) Implementing school health programmes in six districts - Adilabad, Khammam, Nagarkurnool, Asifabad, Mahbubnagar, Jayashankar Bhupalpally. |
| Secondary (Early detection) | a) Population-based screening of all men and women above 30 years for three major cancers (oral, breast and cervical) has been operational since 2018 as a part of comprehensive Primary Health Care Programme, NPCDCS;  b) Provision of the NCD – ASHA app (available in Telugu and Hindi), which enables community-health-workers to enumerate population, complete the community-based assessment checklist and mobilize all individuals above 30 years for screening;  c) Training of ANMs at SC and medical officer at PHC, CHC, district hospital for cancer screening; |
| Tertiary (diagnosis confirmation and treatment) | a) Regional cancer centre (Mehdi Nawaj Jung (MNJ) Institute of Oncology) with state-of-the-art facilities for diagnosis, staging, treatment initiation and completion & palliative care;  b) Tertiary cancer care center at Nizam Institute of Medical Sciences (tertiary cancer care centre) and other private hospitals in Hyderabad.  c) In other districts, Aarogyasri-empanelled hospitals largely cater to the diagnostic care needs of patients.  d) Govt. proposed to set-up a new L1 greenfield comprehensive cancer care centre: apex centre with facilities for advanced diagnostics, treatment, research, and education) in Hyderabad.  e) Strengthening medical colleges at Adilabad, Nizamabad, Mahbubnagar and Warangal as L2 centres to provide diagnostic and treatment services; |
| Palliative care (symptomatic management and end-of-life-care) | a) Well-established palliative care departments in the existing MNJ-RCC and NIMS Hospital for palliative care,  b) Palliative care unit in the Area hospital in Chevella;  c) Eight palliative healthcare centres have been established for in-patient services, one each in the districts of Adilabad, Janagaon, Yadadri, Warangal urban, Rangareddy, Siddipet, Khammam and Mahbubnagar.  d) 30 mobile units in addition to 110 SCs/HWCs have been delivering home care services.  e) The Government has decided to set up palliative care centres in all districts of the State that would offer both in-patient (consist of eight to ten beds with in-patient facility located in the premises of the local government hospital) and home care services (offered in a radius of 30-40 kms). Vehicles titled as “AALANA” have been approved by the health ministry for providing palliative care services.  f) Training of medical officer at district hospital for palliative care;  g) Two-day training in palliative care was planned for 1000 staff including PHC Medical Officers, nurses, paramedical workers and other health staff |
| **State governments plan for the fiscal year 2020-21** | |
|  | As part of the supplementary programme implementation plan (PIP) proposed for 2019-20):  a) To establish district NCD cells: TS received administrative approval for establishment of district NCD cells at Hyderabad, Mulugu and Narayanpet at Rs. 5 lakhs for each of the three districts.  b) Optimize services of experts: The state proposed to optimize services of existing gynaecologists and surgeons for provisional diagnosis and clinical management at cancer day care centres.  c) To establish mammography units and video colposcopy units: To this end, Rs.295 lakhs have been approved for the financial year 2020-21 to establish mammography units and video colposcopy units in 10 District Hospitals of ten erstwhile district headquarters.  d) To implement school health programmes in six districts: Telangana State received NHM administrative approval of Rs. 420 lakhs for implementing school health programmes in six districts - Adilabad, Khammam, Nagarkurnool, Asifabad, Mahbubnagar, Jayashankar Bhupalpally.  e) To establish an L2 centre District Hospital, Khammam: Rs.1,273 lakhs had been approved for establishing L2 centre at District Hospital, Khammam for providing diagnostic, follow-up-treatment and palliative care for cancer patients.  f) To establish 16 district diagnostics hubs: 16 district hubs were approved (with Rs. 4.5 lakhs for renovation and Rs. 44 lakhs for purchase of equipment at each hub) to implement free diagnostics and reduce out-of-pocket expenditure.  g) To procure drugs and supplies: The amount proposed and approved for procurement of drugs and supplies under NPCDCS stands at Rs. 2,627.83 lakhs.  h) Team-based incentive to ASHAs and ANMs to implement population-based NCD screening: For implementing population-based NCD screening in sub-centres, state government has committed to provide Rs.15,000 as team-based incentive to ASHAs and ANMs involved in screening at each sub-centre or PHC.  i) To train healthcare workers in palliative care: Two-day training in palliative care was planned for 1000 staff including PHC Medical Officers, nurses, paramedical workers and other health staff.  j) IEC and BCC activities: Apart from folk-media performances (three programmes in each of the 683 PHCs) for all National Health Mission interventions, government plans to implement innovative IEC/BCC strategies (mobile-based solutions, social media and engagement of youth) under both National Tobacco Control Programme (NTCP) and NPCDCS.  k) Orientation sessions: Orientation sessions at the District Tobacco Control Centres for law-enforcers was also approved under NTCP. |
| **Best practices in planning and delivering cancer care services** | |
| Public | a) District level coordination committees formed for public orientation and implementation of COTPA act.  b) Teleconsultation with specialists arranged in certain urban PHCs for suspected cases and screen-positives before referral.  c) Palliative care team at district level: Physician, physiotherapist, (sometimes, AYUSH medical officer), staff-nurses, ANMs, helpers, driver;  d) Facility-based and home-based palliative care services for - Maintenance therapy / Supportive care;  e) Emotional support to allay fear and anxiety;  f) Counselling early-stage cases to continue treatment; |
| Public-private partnership | a) Screening by ANMs and staff-nurses placed at a district hospital  b) Continuous counselling of eligible asymptomatic women  c) Community-mobilization - benefits of screening explained in simple terms  d) Treatment of cervical pre-cancerous lesions (Cryotherapy, thermal coagulation) |
| Non- Governmental Sector | a) Awareness sessions on risk factors;  b) Target population: Adolescents in government schools, children attending NCC camps  c) School-based screening programmes in rural areas  d) Instant monitoring – Cluster-based feedback  e) Diagnostic investigations at subsidized prices  f) Facilitation of therapeutic care in liaison with civil-servants, health department officials  g) Plan to provide dietary supplements for paediatric cancer patients  h) Financial support for adult patients to meet direct non-medical costs |
