# Supplementary material for: Projected cancer burden, challenges, and barriers to cancer prevention and control activities in the state of Telangana
Source: PLoS One. 2023 Jul 14;18(7):e0278357. doi: 10.1371/journal.pone.0278357 (PMC10348541; doi:10.1371/journal.pone.0278357)
Supplement: S1 Appendix — (PDF) [file pone.0278357.s006.pdf]

S1 Appendix: The Telangana Cancer Control Study Group

Hemant Mahajan, MD; Neha Reddy M, MPH; NG Marina Devi, MPH**,** Usha Rani Poli, DNB; M Jayaram, MD; Shailaja Tetali, PhD; GVS Murthy, MD: *from the Indian Institute of Public Health, Hyderabad, India*.

Oncology experts:

Chinta Sanjeeva Kumari, MD (MNJIO & RCC, Hyderabad, India); Raghunadharao Digumarti, DM; (KIMS-ICON Hospital, Visakhapatnam, India); Mahadev Potharaju, MD (Apollo Hospitals, Chennai, India); Jagannath Palepu (Lilavathi Hospital, Mumbai); Leela Digumarti, MD (KIMS-ICON Hospital, Visakhapatnam, India); Mounika Boppana, DM (Medical Oncologist, Krishna Institute of Medical Sciences, Hyderabad, India); Santosh Kumar Chaturvedi, MD (National Institute of Mental Health & Neurosciences, Bangalore, India); Gayatri Palat, DNB (MNJIO & RCC, Hyderabad, India); Srinivas Chakravarthy Gummaraju, DM (Apollo Cancer Hospital, Hyderabad, India); Vijay Anand Reddy, MD (Apollo Cancer Hospital, Hyderabad, India).
